# Partially Purified Extracts of Sea Anemone* Anemonia viridis* Affect the Growth and Viability of Selected Tumour Cell Lines

**DOI:** 10.1155/2016/3849897

**Published:** 2016-09-20

**Authors:** Matteo Bulati, Alessandra Longo, Tiziana Masullo, Sara Vlah, Carmelo Bennici, Angela Bonura, Monica Salamone, Marcello Tagliavia, Aldo Nicosia, Salvatore Mazzola, Paolo Colombo, Angela Cuttitta

**Affiliations:** ^1^Istituto di Biomedicina e di Immunologia Molecolare, Consiglio Nazionale delle Ricerche, Via Ugo La Malfa 153, 90146 Palermo, Italy; ^2^Laboratory of Molecular Ecology and Biotechnology, National Research Council, Institute for Marine and Coastal Environment (IAMC-CNR), Detached Unit of Capo Granitola, Torretta Granitola, 91021 Trapani, Italy

## Abstract

In the last few years, marine species have been investigated for the presence of natural products with anticancer activity. Using reversed phase chromatography, low molecular weight proteins were fractionated from the sea anemone* Anemonia viridis*. Four different fractions were evaluated for their cytotoxic activity by means of erythrocyte haemolysis test, MTS, and LDH assays. Finally, the antiproliferative activities of three of these fractions were studied on PC3, PLC/PRF/5, and A375 human cancer cell lines. Our analysis revealed that the four fractions showed different protein contents and diverse patterns of activity towards human PBMC and cancer cell lines. Interestingly, fractions III and IV exerted cytotoxic effects on human cells. Conversely, fractions I and II displayed very low toxic effects associated with antiproliferative activities on cancer cell lines.

## 1. Introduction

About one-half of the total global biodiversity is represented by marine organisms producing a potent chemical arsenal which enables them to survive in the harsh marine environment. Hence, marine organisms have been considered the largest reservoir of natural molecules to be evaluated for drug activity [1, 2]. It is also well known that invertebrates are able to produce a considerable number of defence molecules including lytic enzymes, antiadhesive factors, and bioactive compounds [3–5]. Several studies have shown the potential pharmacological properties of these molecules which exert a wide range of biological functions such as antioxidant, antihypertensive, antimicrobial, immunomodulatory, antithrombotic, and hypocholesterolemic effects [6–9]. Moreover, peptides from marine organisms have been reported to induce apoptosis and inhibit* in vitro* cell proliferation in different cancer cell lines [10–14].

Resistance to conventional chemotherapeutic drugs and the absence of fully effective treatments led to the introduction of innovative natural compounds for cancer treatment [15]. Among marine organisms, cnidarians represent an ancient group of venomous animals [16], which are specialized in toxins production and delivery. Members of the Anthozoa class are known to exploit an articulated chemical arsenal and recently it has been estimated that at least 250 compounds have been purified from animals of this class [17, 18]. In addition, it has also been shown that venom components may have also therapeutic applications because of their effects in inflammation and neuronal diseases [19, 20]. Thus, the ability of these animals to produce neurotoxins, pore-forming toxins, and venoms is well documented. Moreover, it has been recently demonstrated that products isolated from these species perform other interesting biological functions that have important implications for health, thus, creating novel therapeutic options [10–14].

Biomedical properties of sea anemones extracts or mucus have been reported for* Paracondactylis indicus*,* Paracondactylis sinensis*,* Heteractis magnifica*,* Stichodactyla haddoni* [21],* Stichodactyla helianthus* [22], and* Actinia equina*, respectively [23]. Cytotoxicity, analgesic, and antibutyrylcholinesterasic activities have been described for venom from Mediterranean jellyfish* Pelagia noctiluca* [24, 25]. Moreover, crude extracts from jellyfish* Aurelia aurita* have been reported to exhibit anticoagulant properties [26], while cytotoxic and cancer chemopreventive properties have been described in extracts from shallow water hard corals [27].

Thus, growing bioprospecting efforts were made for screening of novel anticancer agents [17]. To date the sea anemone* A. viridis*, a member of the Anthozoa class, has been evaluated for the production of neurotoxins, protease inhibitors, and antimicrobial peptides [18, 28, 29]. However data reporting the effects of sea anemone extracts on cell growth arrest or proliferation are still lacking. In the present work, crude extracts from* A. viridis* were used in order to explore their potential use as a source of bioactive compounds. Protein extracts were enriched in low molecular weight species and four fractions (named herein I to IV) were collected. Cytotoxic and viability assays were performed on human erythrocytes and PBMC, while antiproliferative effects were analysed on PC3, PLC/PRF/5, and A375 cancer cell lines. Evaluation of the proliferation rate in response to fractions exposure suggests possible biotechnological and biomedical applications.

## 2. Materials and Methods

### 2.1. Sample Collection and* A. viridis* Crude Extract Preparation

Specimens of* A. viridis* were collected from the Capo Granitola Coast (Torretta Granitola, Trapani, Italy) in the south of Sicily and maintained in Millipore Filtered Sea Water (MFSW) at 18 ± 1°C with a 12 h : 12 h light : dark photoperiod as reported in [30]. Water quality parameters (pH, salinity, nitrate, and ammonium levels) were monitored with kits to measure the water quality of the aquarium. Then, the bodies of the animals were separated from tentacles and homogenated at 4°C in a buffer containing 20 mM Tris-HCl pH 7.6, 20 mM NaCl, and 8 mM EDTA. After 2 cycles of centrifugation at 10,000 RPM for 20 min at 4°C, the clarified supernatants were collected and stored at −80°C until use.

### 2.2. Separation and Characterization of the Four Fractions

Sep-Pak C18 Plus Short Cartridge (Waters Corporation, MA, USA) was used to fractionate the* A. viridis* extract. Briefly, a C18 Cartridge was first washed with three different solvents: Methanol, WFI for cell culture (Gibco, Milan, Italy), and a water solution of 0.1% trifluoroacetic acid (TFA) (Sigma Aldrich, Milan, Italy). The crude extract was centrifuged at 3000 RPM for 10 min. Then, the supernatant was collected and diluted 1 : 10 with a solution of Trizma (10 mM)/NaCl (20 mM), acidified with 0.1% TFA, at pH 7.4. The extract was then injected at a flow rate of 2.50 mL/min. At the end of this step, the eluted fraction was discarded and four successive elutions were performed on the solid phase remaining in the column. In particular, 15%, 30%, 45%, and 60% Acetonitrile in acidified water solutions were used to elute the solid phase bound molecules (flow rate of 2.50 mL/min). The four fractions were collected, lyophilized, and purified from Acetonitrile in a Speed Vac SC100 (Savant) overnight. The lyophilic fractions were then reconstituted in 1x PBS pH 7.4 w/t Ca^++^ and Mg^++^ (Gibco, Milan, Italy). The Bradford protein assay (BioRad, Milan, Italy) was used in order to assess the protein concentration in the samples. To define the composition of the four fractions, an aliquot of each preparation was analysed by RP-HPLC on a LiChroCART 250-4 HPLC-Cartridge. Components were eluted and monitored at 280 nm using water (mobile phase A) and Acetonitrile (mobile phase B), with a linear gradient of Acetonitrile (0%–100% v/v) over 30 min.

Finally, to quantify the level of endotoxins (Lipopolysaccharide, LPS) present in the four fractions, Limulus Amebocyte Lysate (LAL) gel-clotting test (Lonza Group LTD) was carried out according to the manufacturer's instructions.

The molecular mass of the active peaks in fraction II was determined by matrix-assisted laser desorption/ionization time-of-flight mass spectrometry with an Ultraflex TOF/TOF (SYMBIOSIS, part of Biopharm GmbH).

### 2.3. Peripheral Blood Mononuclear Cell Preparation

Peripheral Blood Mononuclear Cells (PBMC) from two donors were isolated from heparinised venous blood by density gradient centrifugation using Ficoll-Paque Plus (GE Healthcare Life Sciences, Milan, Italy). PBMC were resuspended in RPMI 1640 medium (Gibco, Milan, Italy) supplemented with 10% foetal bovine serum (FBS, Invitrogen-Gibco, Milan, Italy), 1% antibiotic (penicillin 100 U/mL, streptomycin sulfate 100 mg/mL) (Invitrogen-Gibco, Milan, Italy), 1% nonessential amino acids (AANE-Euroclone), and 1% sodium pyruvate (1 mM, Euroclone) and seeded to a specific concentration for each assay.

### 2.4. Hemolysis Test

To test the potential haemolytic effect of the four fractions (15%, 30%, 45%, and 60% Acetonitrile), an* in vitro* haemolysis assay was employed as previously reported [1]. Briefly, 100 *μ*L of a 7% human erythrocyte solution was incubated in triplicate with 100 *μ*L of increasing concentrations (from 1 *μ*g/mL to 200 *μ*g/mL) of each of the four fractions for 60 min at room temperature. References to 100% and 0% haemolysis were made by incubating the erythrocyte suspension with 100 *μ*L of 1% Triton X-100, as a positive control, or 100 *μ*L of 1x PBS pH 7.4 (Gibco, Milan, Italy), as a negative control, respectively. After incubation, the tubes were centrifuged at 3000 RPM for 2 min, and 100 *μ*L aliquots of the supernatants were transferred to 96-well microtiter plates (Corning, Milan, Italy) and analysed at 405 nm using an Imark Plate Reader (BioRad, Milan, Italy). The percentage of haemolysis was determined as described: % of haemolytic activity = {[OD (405 nm) tested sample − OD (405 nm) negative control]/[OD (405 nm) positive control − OD (405 nm) negative control]}  × 100.

### 2.5. Cell Viability Test

Cell viability was determined* in vitro* by (3-(4,5-dimethylthiazol-2-yl)-5-(3-carboxymethoxyphenyl)-2-(4-sulfophenyl)-2H-tetrazolium) MTS assay, using the CellTiter 96® Aqueous One Solution Cell Proliferation Assay kit (Promega, USA). PBMC were prepared from venous blood from two donors as described above and were seeded at the concentration of 15 × 10^4^ cells/well in a 96-well plate (Corning, Milan, Italy), in triplicate. Each well was treated with different concentrations (1 *μ*g/mL, 5 *μ*g/mL, 10 *μ*g/mL, 50 *μ*g/mL, 100 *μ*g/mL, and 200 *μ*g/mL) of each of the four fractions and incubated at 37°C and 5% CO_2_ for 24 h or 48 h. At the defined time point, 20 *μ*L of MTS solution was added, and the cells were further incubated in the same condition for an additional 5 hrs. The reading was performed at 490 nm through the Imark Plate Reader (BioRad). The percentage of viability was determined as described: % of vitality = [OD (490 nm) tested sample/OD (490 nm) negative control] × 100.

### 2.6. Cell Culture

For the* in vitro* assays, three different epithelial tumour cell lines were used. The human prostate adenocarcinoma cell line (PC3), human liver hepatoma (PLC/PRF/5), and human melanoma cell line (A375) were purchased from the American Type Culture Collection (ATCC, Rockville, MD, USA). PC3, PLC/PRF/5, and A375 were grown in a RPMI 1640 (Gibco, Milan, Italy) formulation supplemented with 10% foetal bovine serum (FBS) (Gibco, Milan, Italy), 1% antibiotic (penicillin 100 U/mL, streptomycin sulfate 100 mg/mL) (Gibco, Milan, Italy), 1% nonessential amino acids (Euroclone, Milan, Italy), and 1% sodium pyruvate (Euroclone, Milan, Italy), at 37°C in a humidified CO_2_ atmosphere (5%). PC3 and PLC/PRF/5 cells were cultured in 100 mm × 20 mm dishes (Corning, Milan, Italy), while A375 were cultured in 25 cm^2^ tissue culture flasks (Corning, Milan, Italy) and maintained in a monolayer culture. Experiments were performed when cells reached about 80–85% confluence.

### 2.7. Cytotoxicity-Lactate Dehydrogenase (LDH) Assay

The cytotoxicity of the four Acetonitrile fractions (15%, 30%, 45%, and 60%) was quantified by measuring the amount of total lactate dehydrogenase (LDH) released by cells using a colourimetric assay (Roche Applied Science, Milan, Italy) on three cancer cell lines (PC3, PLC/PRF/5, and A375). Cancer cell lines were plated at the concentration of 1-2 × 10^3^ cells/well in 96-well plates (Corning, Milan, Italy) and incubated overnight at 37°C in an atmosphere of 5% CO_2_ to allow their adhesion. Then cells were treated with different concentrations of the four Acetonitrile fractions (10 *μ*g/mL, 50 *μ*g/mL, 100 *μ*g/mL, and 200 *μ*g/mL, final volume 100 *μ*L) and incubated at 37°C and 5% CO_2_ for 24 h or 48 h, using 2% of FBS on RPMI 1640 culture medium. Before harvesting, 10% Triton X-100 was added to the well as positive control (100% cell lysis), with RPMI 1640 culture medium from untreated wells as negative control. At the defined time point, 50 *μ*L of the supernatants was collected. Fifty *μ*L of LDH assay reagent was added to supernatants and incubated for up to 30 min at room temperature in the dark. The absorbance of samples was measured at 490 nm, with a reference reading at a wavelength above 600 nm on a BIOTEK Synergy HT apparatus. The cytotoxicity percentage was determined as described: % of cytotoxicity = [OD (490 nm) tested sample − OD (490 nm) untreated sample]/[OD (490 nm) positive control − OD (490 nm) untreated sample] × 100.

### 2.8. Cell Proliferation

The proliferation study of the cancer cell lines was performed* in vitro* by BrdU proliferation assay, using the Cell Proliferation ELISA BrdU (colourimetric) kit (Roche). PC3, PLC/PRF/5, and A375 were plated at the concentration of 1-2 × 10^3^ cells/well in 96-well plates (Corning, Milan, Italy) and incubated overnight at 37°C in an atmosphere of 5% CO_2_ to allow their adhesion. Then cells were treated, with different concentrations of the 15%, 30%, and 45% Acetonitrile fractions (10 *μ*g/mL, 50 *μ*g/mL, 100 *μ*g/mL, and 200 *μ*g/mL, final volume 100 *μ*L) and incubated at 37°C and 5% CO_2_ for 24 h or 48 h. Two hours before the end-time point, cells were treated with 10 *μ*L of BrdU labelling solution and put back into the incubator until the end of 24 h or 48 h. After this treatment, the medium was removed and cells were incubated for 30 min with 200 *μ*L of FixDenat solution at room temperature to fix the cells and denature the DNA and then for 90 min with anti-BrdU-POD solution. Antibody conjugates were removed by washing with 200 *μ*L of washing buffer. After incubation with a 3,3′,5,5′-tetramethylbenzidine (TMB) substrate for 15 min, immune complexes were detected by measuring the absorbance at 450 nm. The reading was performed using the WALLAC 1420VICTOR^2^ reader (Perkin Elmer). The proliferation percentage was determined as described: % of proliferation = [OD (450 nm) tested sample/OD (450 nm) negative control] × 100.

### 2.9. Acridine Orange Staining

PC3 and A375 cells, seeded at low density (2000 cells per well) onto microscope cover slips in 12-well culture plates, were grown for 48 hrs in presence or absence of 50 *μ*g/mL fraction II. After the incubation period, the medium was removed; cells were then fixed in 3.7% formaldehyde and stained with 10 *μ*M Acridine Orange in PBS for 5 minutes. After 6 washes in PBS, cells were observed at fluorescence microscope (Leica, DFC450C).

### 2.10. Measurement of Cellular ROS Production

Cellular ROS production was measured using a DCFDA assay kit according to manufacturer's protocol (Abcam ab113851). PC3 and A375 cells were grown on 96-well plates. When reached confluence, cells were washed twice with PBS and treated with 25 *μ*M DCFDA in essential medium with 10% FBS at 37°C incubator for 30 min. After PBS washing, cells were exposed to 50 *μ*g/mL fraction II for 48 hours and fluorescence plates were measured in 96-well at 485 nm excitation and 535 nm emission using Tecan Infinite M200 Multimode Microplate Reader (Tecan US, Inc., Morrisville, NC). Background fluorescence was subtracted from each value. All experiments were done in triplicate.

### 2.11. Apoptosis Evaluation by Enzymatic Assay

PC3 and A375 cells were grown for 48 hrs in presence or absence of 50 *μ*g/mL fraction II. Cells were enzymatically detached using Trypsin-EDTA 1x (Sigma) solution and centrifuged at 1000 rpm for 5 min.

Pelleted cells were lysed with 70 *μ*L of 1% Triton X-100 solution in PBS, incubated 10 min at room temperature, and successively centrifuged at 10,000 rpm for 10 min. The amount of protein extract in the supernatant medium was quantified using Bradford microassay method (BioRad, Segrate, Milan, Italy).

PC3 and A375 cells extracts corresponding to 20 *μ*g protein extracts were used to detect the presence of activated caspases 3/7/8, using Ac-Asp Glu-Val-Asp-MCA peptide (Pepta Nova, 380 Peptide Institute). The degree of caspase activation was quantified by spectrofluorimetric readings using Spectra Max Gemini EM-500 (Molecular Devices) and elaborated by SoftMax Pro 5.2 software.

Untreated PC3 and A375 cells were used as negative control while cells treated with Doxorubicin (DXR, Ebewe Pharma) at 5 *μ*M for 24 h were used as positive control.

### 2.12. Statistical Analyses

Data are expressed as the mean ± SD of the percentage of haemolysis (haemolytic assay), viability (MTS assay), or proliferation (BrdU assay) which are expressed as the mean of the percentage of cytotoxicity (LDH assay). All statistical analyses were made with GraphPad InStat 3 using nonparametric ANOVA test to compare two independent groups of data. Differences are considered significant when a *p* value < 0.05 was obtained by comparison between untreated cells and cells stimulated with different concentration values of the 15%, 30%, 45%, and 60% Acetonitrile fractions of* A. viridis* body extract.

## 3. Results

### 3.1. Characterization of the* A. viridis* Extracts

*A. viridis* whole extract showed various bands ranging from hundreds to less than 3 kDa when resolved by SDS-PAGE (data not shown). Since it is known that most biological activities are usually found in the lower molecular weight fraction, we enriched samples using a C18 reversed phase liquid chromatography. A discontinuous gradient, corresponding to Acetonitrile 15%, 30%, 45%, and 60%, was used to elute molecules and four different fractions were collected and herein named as fractions I (Acetonitrile 15%), II (Acetonitrile 30%), III (Acetonitrile 45%), and IV (Acetonitrile 60%). Fractions were lyophilized to remove any Acetonitrile trace and reconstituted in PBS w/o Ca^2+^ and Mg^2+^ pH 7.4.

**Figure 1:**
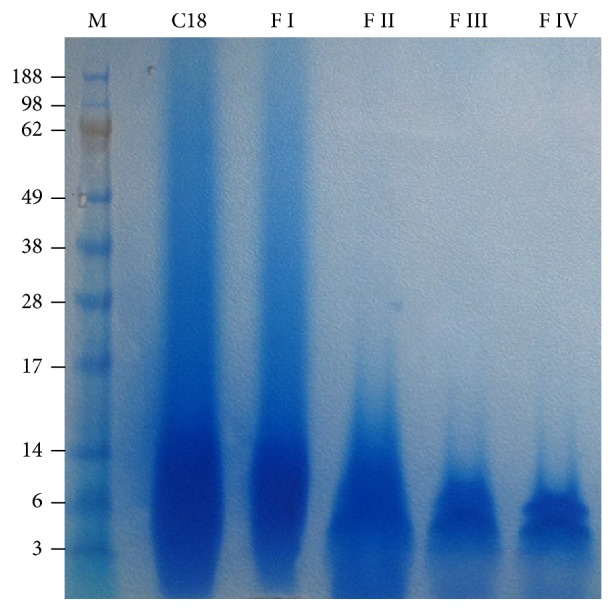
SDS-PAGE of recovered proteins and peptides by RP-LC from* A. viridis*. Lane M indicates molecular weight marker (SeeBlue Plus Prestained ladder, Invitrogen). At least 10 *μ*g of total protein extract recovered after RP-LC separation (C18) or after discontinuous Acetonitrile gradient (F I–IV) was loaded on NuPAGE Novex 4–12% Bis-Tris Gel.

In all fractions, the absorbance at 280 nm suggested the presence of proteins and their amount increased until 30% Acetonitrile was used and then declined (data not shown). Corresponding protein concentrations are reported in Table 1. All fractions were also separated on SDS-PAGE to confirm the enrichment in low molecular weight proteins (Figure 1).

Moreover, in order to exclude any cytotoxic effects exerted by LPS in subsequent tests, fractions were further analysed by LAL test for the presence of endotoxins. None of the fractions displayed detectable amount of LPS (limit of detection ≤ 0.003 ng LPS/*μ*g of fraction).

The electrophoretical separation for all the fractions resulted in a similar pattern, which was better resolved in reversed phase-high performance liquid chromatography (RP-HPLC).

**Figure 2:**
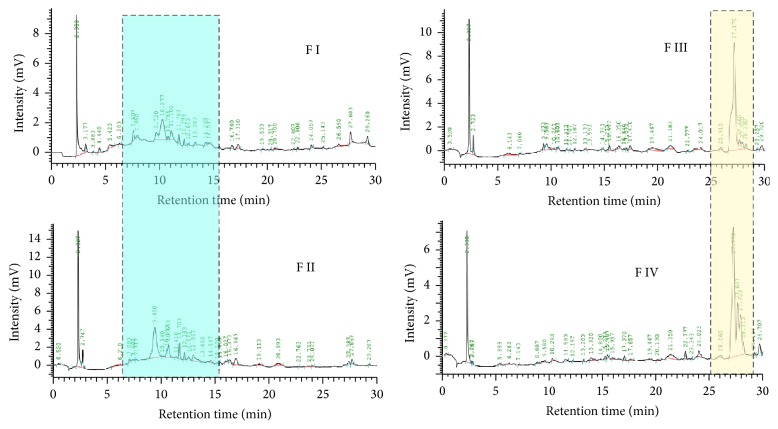
RP-HPLC of the* A. viridis* fractions. *x*-axis shows retention time, while *y*-axis shows intensity of the read-out.

Fractions I and II showed similar fractionation plots as revealed by the presence of common molecular species, especially in the range of 7–15 minutes of retention time (Figure 2). This situation resembled the overlapping patterns observed for fractions III and IV, which share main peaks with retention times around 27 minutes.

### 3.2. Evaluation of Cytotoxic Activity on Human Erythrocytes and PBMC

Analysis of the different HPLC plots raises the possibility that compounds from the fractions I-II and III-IV may exert different effects on selected human cells. Thus, in order to explore the effects of* A. viridis* protein extracts, the cytotoxic activities of all the fractions were evaluated on human erythrocytes and PBMC.

The haemolytic activity of the four fractions on human erythrocytes is reported in Table 2.

Fractions I, II, and III did not exhibit significant haemolytic activity on human erythrocytes, even at highest dosage (200 *μ*g/mL). Conversely, fraction IV exerted haemolytic activity which reached a 28.4% value at highest dosage (Table 2).

**Figure 3:**
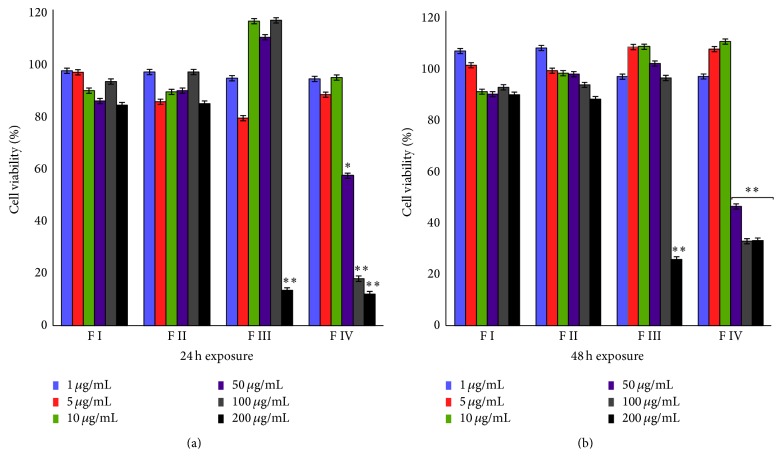
Evaluation of the viability percentage of PBMC by MTS assay after 24 h (panel (a)) or 48 h (panel (b)) of exposure with 1 *μ*g/mL, 5 *μ*g/mL, 10 *μ*g/mL, 50 *μ*g/mL, 100 *μ*g/mL, and 200 *μ*g/mL of each fraction. Data are expressed as mean ± SD of two different experiments in triplicate. All statistically significant differences were evaluated with ANOVA nonparametric test, comparing the untreated sample with each of the different concentrations of the four fractions, and are displayed at the top of the corresponding histogram when present (^*∗*^ *p* < 0.05; ^*∗∗*^ *p* < 0.01).

To confirm the cytotoxic evidences exerted by fractions, viability assays on human PBMC were carried out. Cells from two donors were exposed at increasing concentrations (from 1 *μ*g/mL to 200 *μ*g/mL) of the different fractions for 48 hours. Results reported in Figure 3 show the viability of PBMC at 24 h (panel (a)) and 48 h (panel (b)).

Fractions I and II did not affect cell viability at any concentration even after 48 h. PBMC exposure to fraction III for 24 and 48 hours resulted in a similar reduction of cell viability only at highest concentration. Conversely, a significant reduction in cell viability was observed when PBMC were exposed to increasing concentrations of fraction IV. Viability was reduced after exposure to 50 and 100 *μ*g/L fraction IV and the lowest cell viability was detected at 200 *μ*g/mL.

### 3.3. Cytotoxic Effects on Cancer Cell Lines

**Figure 4:**
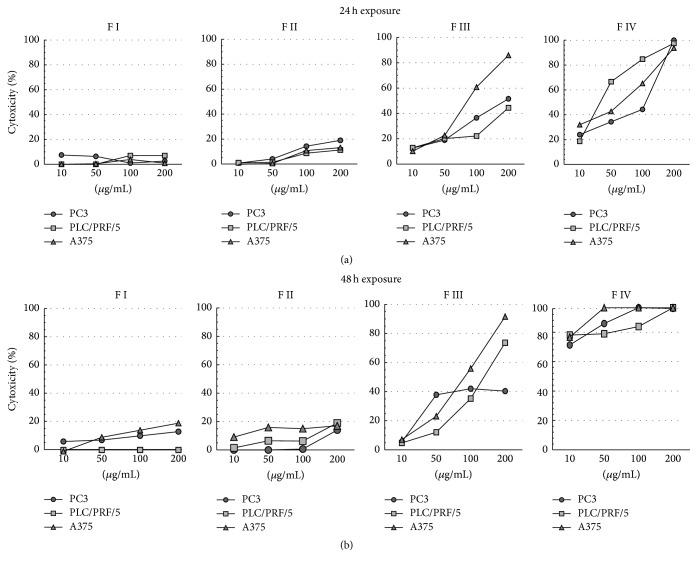
Evaluation of the cytotoxicity percentage of fractions I, II, III, and IV on the three cancer cell lines, PC3, PLC/PRF/5, and A375, after 24 h (a) or 48 h (b) of treatment with or without (untreated cells) 10 *μ*g/mL, 50 *μ*g/mL, 100 *μ*g/mL, and 200 *μ*g/mL of each fraction. Data are expressed as the mean of an experiment in triplicate.

To further characterize the four fractions, their cytotoxic activity was tested on three different cancer cell lines (PC3, PLC/PRF/5, and A375). LDH release was measured in culture medium after exposure to 10, 50, 100, and 200 *μ*g/mL of each fraction and different results were obtained. As shown in Figure 4, exposure to fractions I and II did not affect cell survival since any appreciable release of LDH from the three cancer cell lines was measured even after 48 h of treatment. Conversely, exposure to fractions III and IV resulted in cellular damage since a dose-dependent release of LDH was detected. The measured cytotoxicity reached value about 50% in PC3 and PLC/PRF/5 cells and 90% cytotoxicity in A375 melanoma cancer cells. Additionally, a further increase in cytotoxic effects on PLC/PRF/5 cell lines was observed after 48 h of treatment.

Exposure to 100 *μ*g/mL fraction IV induces 100% cytotoxicity at 24 h in all three cell lines and a severe cytotoxicity was detected after 48 h even at lower concentrations.

### 3.4. Antiproliferative Effects on Cancer Cell Lines

The dramatic cytotoxic effects which occurred among all the cell lines previously exposed to fraction IV prompted us to exclude fraction IV from further investigations and to focus only on properties of fractions I, II, and III.

**Figure 5:**
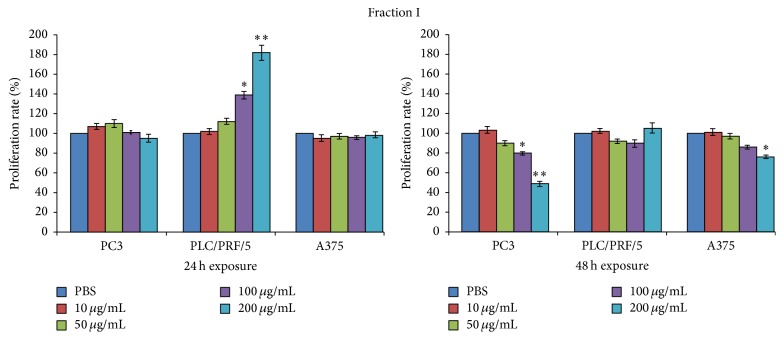
Evaluation of the proliferation rate percentage (incorporation of BrdU) of PC3, PLC/PRF/5, and A375 cancer cell lines after 24 and 48 h of culture with or without 10 *μ*g/mL, 50 *μ*g/mL, 100 *μ*g/mL, and 200 *μ*g/mL of the fraction I. Data are expressed as the mean ± SD of three different experiments in triplicate. All statistically significant differences were evaluated with ANOVA nonparametric test, comparing the untreated sample with each of the different concentrations of the three fractions, and are displayed at the top of the corresponding histogram when present (^*∗*^ *p* < 0.05; ^*∗∗*^ *p* < 0.01).

**Figure 6 fig6:**
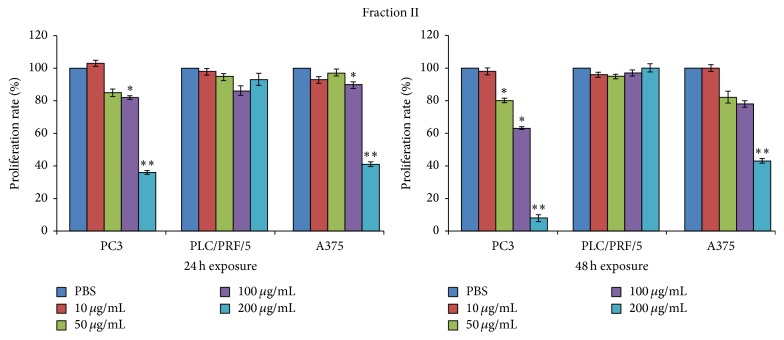
Evaluation of the proliferation rate percentage (incorporation of BrdU) of PC3, PLC/PRF/5, and A375 cancer cell lines after 24 and 48 h of culture with or without 10 *μ*g/mL, 50 *μ*g/mL, 100 *μ*g/mL, and 200 *μ*g/mL of the fraction II. Data are expressed as the mean ± SD of three different experiments in triplicate. All statistically significant differences were evaluated with ANOVA nonparametric test, comparing the untreated sample with each of the different concentrations of the three fractions, and are displayed at the top of the corresponding histogram when present (^*∗*^ *p* < 0.05; ^*∗∗*^ *p* < 0.01).

**Figure 7:**
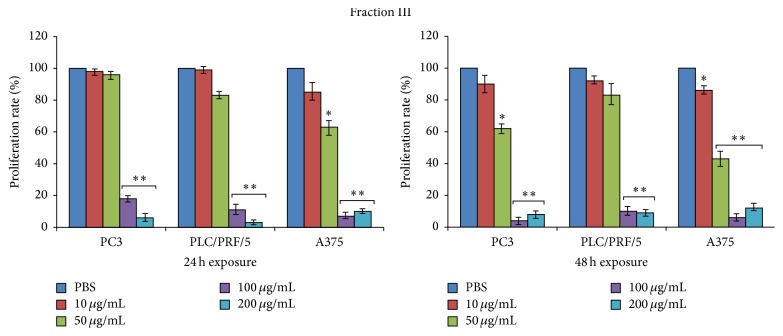
Evaluation of the proliferation rate percentage (incorporation of BrdU) of PC3, PLC/PRF/5, and A375 cancer cell lines after 24 and 48 h of culture with or without 10 *μ*g/mL, 50 *μ*g/mL, 100 *μ*g/mL, and 200 *μ*g/mL of the fraction III. Data are expressed as the mean ± SD of three different experiments in triplicate. All statistically significant differences were evaluated with ANOVA nonparametric test, comparing the untreated sample with each of the different concentrations of the three fractions, and are displayed at the top of the corresponding histogram when present (^*∗*^ *p* < 0.05; ^*∗∗*^ *p* < 0.01).

Thus, these fractions were tested for their effects on tumour cell lines proliferation. PC3, PLC/PRF/5, and A375 cell lines were exposed to 10 *μ*g/mL, 50 *μ*g/mL, 100 *μ*g/mL, and 200 *μ*g/mL of the three fractions and incorporation of BrdU into newly synthesized DNA strands of actively proliferating cells was measured after 24 and 48 h (Figures 5 [Fig fig6]–7).

Proliferation of PC3 or A375 cancer cell line was not affected by exposure to fraction I during the first 24 hours of exposure (Figure 5); interestingly, a significant increase in PLC/PRF/5 cells proliferation became noticeable at 100 *μ*g/mL and 200 *μ*g/mL. In addition, the overall pattern was maintained at 48 h; however some changes occurred as a significant reduction of proliferation rate on PC3 and A375 cells lines and the loss of the stimulating activity on the PLC/PRF/5 proliferation.

Proliferation of PC3 or A375 cancer cell line was not affected by exposure to fraction II at the lower concentrations, while significant antiproliferative effects were showed at the highest concentrations (Figure 6). However, a moderate still significant reduction in proliferation was measured at 100 *μ*g/mL in PC3 cells. Moreover, PLC/PRF/5 cells were not influenced in their proliferative activity.

Finally, fraction III (Figure 7) seems to exert concentration-dependent antiproliferative effects in all three cell lines, reaching significant values starting from 50 *μ*g/mL in A375 cells and from 100 *μ*g/mL in PC3 and PLC/PRF/5 cells.

As what occurred for the other fractions at 48 h, the reduction in cell proliferation was also boosted in all the three tumour cell lines.

### 3.5. Mass Spectrometric Analysis

**Figure 8:**
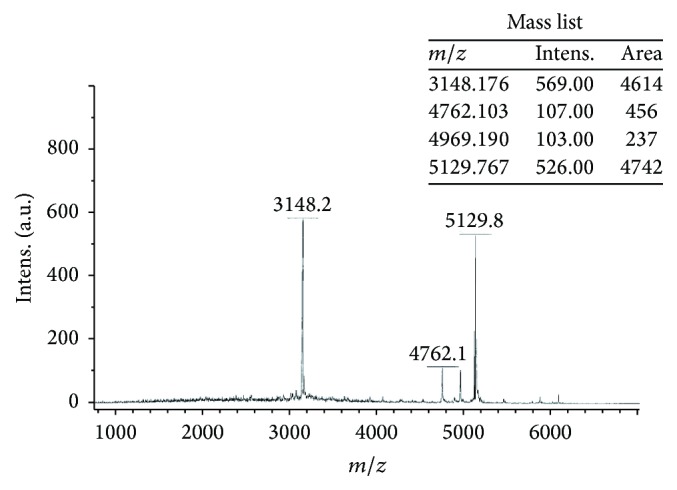
MALDI-TOF mass spectrum of fraction II from* A. viridis*. Four peptides with different molecular weight were detected. Intensity and relative quantity were also reported.

The fraction II, which was found to be active within the 24 hours in absence of significant cytotoxicity, was analysed by MALDI-TOF-MS. The mass spectrometric analysis showed preponderantly the presence of two peptides with molecular weight of 3148.2 and 5129.8 Da. Additionally, two peptides with molecular weight of 4762.1 and 4969.1 Da, present at trace level, were also detected (Figure 8).

### 3.6. Morphological and Enzymatic Analysis for Cell Death

**Figure 9:**
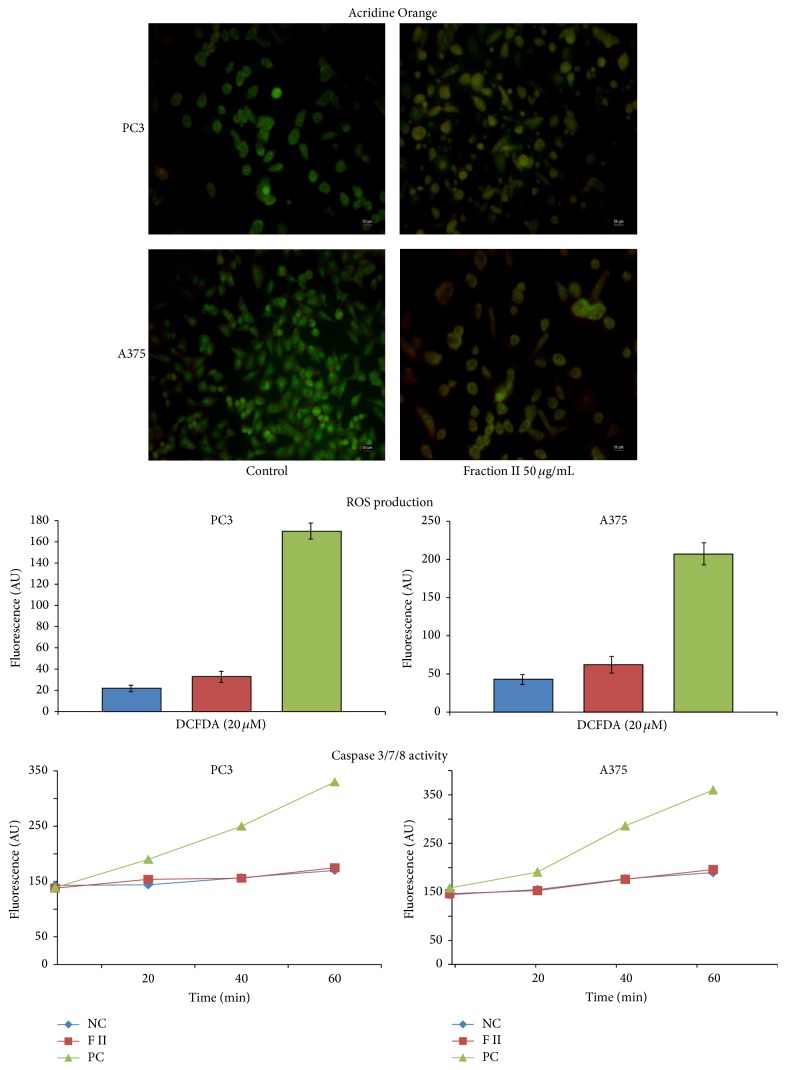
Biological evaluations of fraction II. PC3 and A375 cells were exposed for 48 h to 50 *μ*g/mL. Acridine Orange staining, ROS production, and activation of apoptotic pathways were performed.

To further characterize the effects of sea anemone extracts, a morphological evaluation by fluorescence microscopy analyses was carried out on responsive PC3 and A375 cancer cell lines. Cells were incubated with 50 *μ*g/mL of fraction II for 48 hours and stained with an Acridine Orange solution (Figure 9).

Unexposed PC3 and A375 cancer cell lines do not show any morphological alterations. Conversely, exposure to 50 *μ*g/mL affected cell structures and blebbing of plasma membrane appeared. Moreover, nuclei with condensed or fragmented chromatin were still present. Thus, it could be hypothesized that mechanisms of cell death (apoptosis or necrosis) may occur after exposure to sea anemones extracts.

In order to unveil cellular events leading to the observed cell death, enzymatic assays based on measurements of ROS production and activation of apoptotic pathways were performed.

In particular, the production of intracellular ROS was evaluated monitoring the changes in DCF fluorescence intensity in both control and PC3 and A375 cancer cell lines exposed to 50 *μ*g/mL of fraction II for 48 hours.

No significant differences were observed in control and exposed cells as DCF fluorescence intensity does not increase during the experiments.

Similarly, when the activities of caspases 3, 7, and 8 were analysed, no significant differences were measured in PC3 and A375 cancer cell lines exposed to 50 *μ*g/mL of fraction II for 48 hours.

At different incubation times with the caspase substrate cells' extracts were analysed to determine the amount of fluorescence of each sample. As shown, PC3 and A375 cells did not show any activation of caspases, while time-dependent fluorochrome release was observed for the DXR-treated cells (positive control PC).

## 4. Discussion

It is widely accepted that tumorigenesis is allowed by uncontrolled cellular growth and spreading of abnormal cells. Under physiological conditions, proliferation and apoptosis are balanced. Conversely, cancer cells usually display abnormal proliferation that is not balanced by compensatory cell death mechanisms.

Herein, a screening for cytotoxicity and antiproliferative activity of* A. viridis* was described. The use of C18 reversed phase resin enabled the enrichment in low molecular weight proteins; they were further fractionated by means of discontinuous gradient deriving from a stepwise variation of the Acetonitrile percentage (15%, 30%, 45%, or 60%), and four different fractions were recovered. Haemolysis and viability assays on human PBMC were firstly carried out on all fractions in order to differentiate cytotoxic from antiproliferative actions. Thus, analysis of potential antiproliferative effects was limited to fractions I, II, and III. In particular fractions I and II exerted antiproliferative effects on both the ectoderm derived PC3 and A375 cell lines in absence of significant cytotoxicity, while the endo/mesoderm derived PLC/PRF/5 hepatoma cells were not affected by any dosage. Conversely, fraction III showed both cytotoxic and antiproliferative activities. Interestingly, the HPLC analysis of this fraction revealed a pattern similar to fraction IV which also showed cytotoxic effects.

Consistent with our results and concentrations herein evaluated, other authors reported that fractions from the Mediterranean gorgonian* Eunicella singularis* exhibit antiproliferative properties against A549, HCT15, and MCF7 cancer cell lines in concentrations ranges spanning from 36 to 426 *μ*g/mL exerted lines [31]. Similarly, the exposure to 50 *μ*g/mL of fractions from the sea anemone* Bunodeopsis globulifera* was shown to increase cisplatin cytotoxicity to human lung adenocarcinoma cells inducing a reduction in cell viability of approximately 50% [32].

Interestingly, PC3 and A375 cells are known to express high levels of glutathione-S-transferase-*π* (GST-*π*) and multidrug resistance-associated protein (MRP) which are related to multidrug resistance [33].

It has been shown that extracts from different cnidarians species may affect cell survival by induction of apoptosis [34]. Thus, in order to better understand the activity of fraction II from* A. viridis*, which has been found to be active within the 24 hours without significant cytotoxicity, morphological and enzymatic assays were performed on PC3 and A375 cell lines.

Interestingly, data herein presented suggest that antiproliferative activities of fraction II were associated with induction of cell death machinery as observed after Acridine Orange staining. Additionally, some morphological alterations observed in PC3 and A375 cells after exposure, including blebbing of the membranes, may be associated with mechanisms of programmed cell death [35].

Moreover, data herein reported suggest that fraction II may likely induce cell death without the involvement of ROS and caspases.

Thus, other pathways than those related to ROS and caspases may be activated in PC3 and A375 cells.

Hence, it appears that the Mediterranean sea anemone may be able to produce compounds with pharmacological activities.

## 5. Conclusions

Data herein reported support the hypothesis that peptides from fraction II of* A. viridis*, in addition to cytotoxic activities, are capable of inhibiting proliferation of tumour cell lines. Further characterizations of the most active fractions are under investigation in order to isolate and better purify bioactive compounds exerting these pharmacological activities.

## Figures and Tables

**Table 1:** Protein concentrations in all the fractions by Bradford assays.

Fractions	Concentration (*µ*g/*µ*L)
I	0.7
II	1.4
III	1
IV	0.6

**Table 2:** Percentage of haemolytic activity of *A. viridis* fractions on human erythrocytes.

	Dosage (*µ*g/mL)					
	1	5	10	50	100	200
Fraction I	nd	nd	nd	nd	0.3%	0.3%
Fraction II	nd	0.3%	0.3%	0.3%	0.3%	0.6%
Fraction III	nd	0%	0%	0.3%	0.3%	0.3%
Fraction IV	nd	0%	0%	0.3%	1.2%	28.4%
